# Physical versus psychological social stress in male rats reveals distinct cardiovascular, inflammatory and behavioral consequences

**DOI:** 10.1371/journal.pone.0172868

**Published:** 2017-02-27

**Authors:** Julie E. Finnell, Calliandra M. Lombard, Akhila R. Padi, Casey M. Moffitt, L. Britt Wilson, Christopher S. Wood, Susan K. Wood

**Affiliations:** Department of Pharmacology, Physiology, and Neuroscience, University of South Carolina School of Medicine, Columbia, South Carolina, United States of America; Technion Israel Institute of Technology, ISRAEL

## Abstract

Repeated exposure to social stress can precipitate the development of psychosocial disorders including depression and comorbid cardiovascular disease. While a major component of social stress often encompasses physical interactions, purely psychological stressors (i.e. witnessing a traumatic event) also fall under the scope of social stress. The current study determined whether the acute stress response and susceptibility to stress-related consequences differed based on whether the stressor consisted of physical versus purely psychological social stress. Using a modified resident-intruder paradigm, male rats were either directly exposed to repeated social defeat stress (intruder) or witnessed a male rat being defeated. Cardiovascular parameters, behavioral anhedonia, and inflammatory cytokines in plasma and the stress-sensitive locus coeruleus were compared between intruder, witness, and control rats. Surprisingly intruders and witnesses exhibited nearly identical increases in mean arterial pressure and heart rate during acute and repeated stress exposures, yet only intruders exhibited stress-induced arrhythmias. Furthermore, re-exposure to the stress environment in the absence of the resident produced robust pressor and tachycardic responses in both stress conditions indicating the robust and enduring nature of social stress. In contrast, the long-term consequences of these stressors were distinct. Intruders were characterized by enhanced inflammatory sensitivity in plasma, while witnesses were characterized by the emergence of depressive-like anhedonia, transient increases in systolic blood pressure and plasma levels of tissue inhibitor of metalloproteinase. The current study highlights that while the acute cardiovascular responses to stress were identical between intruders and witnesses, these stressors produced distinct differences in the enduring consequences to stress, suggesting that witness stress may be more likely to produce long-term cardiovascular dysfunction and comorbid behavioral anhedonia while exposure to physical stressors may bias the system towards sensitivity to inflammatory disorders.

## Introduction

Depression affects approximately 7 percent of adults and 11 percent of adolescents, making it one of the leading causes of disability in the United States [1, 2]. In addition to the well-known behavioral deficits, depression has been associated with a 2–3 fold greater risk of developing cardiovascular disease later in life [3, 4] resulting in significant co-morbidity [5, 6]. Similarly, cardiovascular disease is also associated with enhanced risk for developing depression [7, 8]. While this co-morbidity has been well established, the mechanism by which depression can increase the risk of cardiovascular disease and alternatively, cardiovascular disease can enhance the risk of psychopathology remains largely unknown. However, it has been suggested that stress may underlie this co-morbidity as stress has been shown to promote the emergence of both depression [9] and cardiovascular disease [10, 11].

Social stress, such as bullying or abuse, is considered to be one of the most common types of stress individuals face in their lifetime [9]. The resident-intruder paradigm of social defeat is often used to model social stress in rodents as it is well known to result in the emergence of depressive-like behaviors, such as immobility in the forced swim test and reduced sucrose preference [12–16]. Interestingly, social stress in rodents also results in cardiovascular deficits including increased heart rate (HR), blood pressure (BP), and arrhythmias [17–20]. Together, these data suggest that social defeat is highly relevant as a model to study mechanisms involved in depressive-cardiovascular disease co-morbidity as it recapitulates the negative social interactions that may lead to the development of these disorders.

While a major component of social stress often encompasses physical interactions, purely psychological stressors such as witnessing parental violence [21] or viewing images of extreme violence [22] also fall under the scope of social stress. Interestingly, similar to physical forms of social stress, psychological stressors also result in an increase in HR and BP [23] and produce a 2-fold greater risk of developing depression [21], with the frequency of exposure being predictive of symptom severity [22]. Currently, inducing psychological stress in animals is often accomplished by allowing a rodent to observe, or witness, another rodent being subjected to a physical stressor. For example, some models use a witness stress foot-shock paradigm, which forces a rat or mouse to witness a cage mate receiving repeated inescapable foot shocks [24–28]. Use of this paradigm often produces distinct effects in witnesses compared to the foot shock animals, such that witness stressed rats exhibit enhanced sensitivity to nociception [25], cocaine self-administration [28], saccharine preference [27], and hyperactivity in the open field test [26] and elevated plus maze [27] while their foot shocked counterparts do not.

More recently, witness models have emerged that consist of a male observing the resident-intruder model of social defeat stress, thereby allowing for a comparison between physical and psychological exposure to social stress [29–33]. Social defeat stress not only has ethological relevance, as rodents (rats, mice, and prairie voles) naturally exist in a social hierarchy, but also provides face validity to stressors capable of inducing a depressive-like state [14, 34–38]. Only a select few studies report using this social defeat witness stress model, yet it has been shown that this form of witness stress results in the emergence of robust anxiety- and depressive-like behaviors [29–31, 33], and enhanced peripheral immune [32] and neuroendocrine [31] responses, that resemble findings in paired socially defeated animals [33]. However, the cardiovascular and neuroimmune consequences of such a stressor remain largely unknown. Therefore, the focus of this study was to determine if social stress in the form of direct interaction with the aggressive resident produces differential cardiovascular, behavioral, and immune outcomes compared with witnessing such an interaction in male rats. Gaining a better understanding of the distinct neural and cardiovascular changes associated with different forms of social stress provide insight to the unique mechanisms involved in susceptibility to social stress-induced consequences.

## Methods

**Fig 1:**
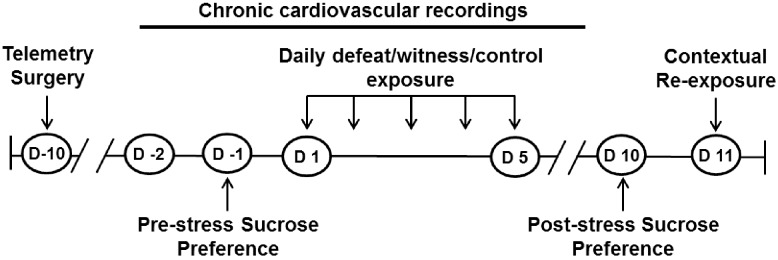
Study design and timeline. Rats were implanted with cardiovascular (CV) radio-telemetric transmitters 10 days prior (D -10) to the start of stress manipulation. Baseline CV measurements were collected for 2 days (D -2—D 0) prior to the start of the daily control, defeat, or witness stress exposure and continued for 3 days post stress/control. Sucrose preference tests were administered 2 days prior to (D -1) and 5 after (D 10) stress manipulations to determine changes in hedonic behavior. On the day of sacrifice (D 11) all rats were exposed to a 15-minute contextual re-exposure.

A brief study timeline is depicted in Fig 1.

### Animals

Male Sprague-Dawley rats (225–250g, intruder, witness, or controls) and Long-Evans retired breeders (650–850g, residents) (Charles River, Wilmington MA) were individually housed for the duration of the study in standard cages with *ad libitum* access to food and water while maintaining a 12-hour light/dark cycle. Care and use of the animals was approved by the University of South Carolina’s IACUC and was in accordance with the NIH Guide for the Care and Use of Laboratory Animals.

### Cardiovascular transmitter surgery

10 days prior to the start of social defeat, witness, or control manipulation, all Sprague-Dawley rats were implanted with intraperitoneal radio-telemetric transmitters that allow for collection of ECG and blood pressure recordings from awake animals (HD-S11-F0 and -F2, Data Sciences Int., St. Paul, MN). Importantly, HD-S11 F2 and F0 transmitters signal via different frequencies to allow for simultaneous recordings of both the intruder and witness animals within the same cage thereby facilitating direct comparison of cardiovascular responses to physical vs. psychological stress. Rats were anesthetized with isoflurane (2.5%) and transmitters were implanted following steps outlined by Data Sciences Int. in the Modified Lead II configuration. Post-surgery analgesia (Flunazine 2.5mg/kg) and supportive therapy of Bacon Softies (Bio-Serv, Flemington, NJ) were administered for 48 hours post-operation.

### Social defeat/Witness stress

This animal model was modified from the version developed by Sial et al. 2016 [33] and Warren et al. 2013 [31] using the rat model of social defeat developed by Miczek [39]. Residents were screened for their level of aggression prior to being included in the study as previously published [12]. Sprague-Dawley rats were randomly assigned into “intruder” (n = 16), “witness” (n = 8), or “control” (n = 12) groups. Intruders were placed into the cage of a novel resident for 15 minutes on 5 consecutive days during the light cycle. Paired witnesses were forced to observe each of these interactions from behind a perforated Plexiglas partition within the resident cage to allow for olfactory, visual, and auditory cues (S1 Fig). Intruders and witnesses were returned to their respective individual home cages daily following the social stress. Control animals were handled briefly for 15 seconds/day, to mimic the duration of witness/intruder handling time, and were not present in the room during social defeat exposures. See Section A of S1 File.

### Cardiovascular telemetry analysis

All cardiovascular and relative activity data were acquired in Dataquest Art (Data Sciences Int., St. Paul, MN) and analyzed in the Ponemah Physiology platform (Data Sciences Int., St. Paul, MN). A schematic of the cardiovascular telemetry system used to obtain the data collected in this study is presented in S2 Fig; also see Section B of S1 File for detailed telemetry methods.

#### 24-hour resting home cage cardiac data analysis

Chronic 24-hours/day telemetry recordings were collected in 5-minute bins/hour for 10 consecutive days. Baseline values for blood pressure (BP) and heart rate (HR) for each rat represent the mean values from data collected for 48 hours prior to defeat/witness/control. Resting BP and HR from the 12-hour dark cycle after stress exposure was calculated as a “change from the aforementioned baseline”.

#### Stress/Control exposure cardiac data analysis

Continuous cardiovascular data were collected during the light phase on days 1 (initial exposure, i.e. acute), 5 (repeated), and 11 (contextual re-exposure 6 days after the final stress/control). Baseline cardiovascular recordings were acquired 30 minutes prior to stress/control manipulation while each rat remained in its home cage. Recordings were continued throughout the 15-minute control/stress exposure on days 1, 5 and contextual re-exposure on day 11. Data were calculated as a change from baseline comparing mean arterial pressure (MAP) and HR against their own resting, home cage baseline. Premature ventricular contractions (PVCs) were identified using the Lambeth Conventions for the investigation of arrhythmias during continuous baseline (5 minutes) and the first 5 minutes of stress exposure on days 1 and 5 of social stress/control and upon context re-exposure (day 11). The blood pressure trace corresponding to each ECG trace was used to confirm that arrhythmic events were paired with a disturbance in blood pressure.

### Sucrose preference

In order to determine the long-term behavioral deficits that result from intruder or witness stress, all animals were subjected to the sucrose preference test 2 days prior to and 5 days after the final stress/control exposure as previously published [12, 13]. Sucrose preference ([volume 1% sucrose/total volume consumed] x 100) was calculated for the first and third hour of the dark period.

### Context re-exposure, sacrifice, and tissue collection

6 days after the final social defeat or control exposure, all rats were subjected to a 15-minute contextual re-exposure: controls were handled and returned to their home cages, while intruders and witnesses were placed into a soiled resident cage on the appropriate side of the Plexiglas partition in the absence of the resident. All rats were immediately sacrificed following the re-exposure via rapid decapitation as this time point is ideal for visualizing stress-induced epinephrine and peripheral cytokine responses in repeatedly stressed rats [40, 41]. Trunk blood, brains, hearts, spleens, and adrenals were collected. Brains were flash frozen in ice-cold isopentane and plasma was separated from trunk blood after centrifugation at 4°C and 3200 rpm. Brains and plasma were stored at -80°C. Adrenals and spleens were cleaned and weighed immediately and atria were dissected away from the ventricles of hearts for the determination of ventricular weights.

### Locus Coeruleus (LC) collection and homogenization

Posterior brains were sliced to the most caudal level of the LC (Bregma -10.20 mm) [42]. At this time bilateral 1x1 mm tissue punches were collected using a 1mm wide tissue biopsy punch (Harvard Apparatus, Holliston, MA) and stored at -80°C. Visual verification of punch placement and accuracy was assessed by viewing Neutral Red (pH 2.8, Fisher Scientific, Waltham MD) stained 30 μm thick pre- and post-punch brain slices with a Leica ATC 2000 Microscope (See S3 Fig). Only LC punches with accurate placement were homogenized as previously described [12] and stored at -80°C.

### High Performance Liquid Chromatography (HPLC)

The effects of intruder vs. witness stress on plasma epinephrine levels was analyzed by HPLC identical to our previous report [12]. All samples were spiked with an internal standard of 100nM dihydroxybenzylamine (DHBA, Sigma-Aldrich, St. Louis, MO), and filtered using an Amicon Ultra 0.5 mL Centrifugal filter with a 50kD molecular weight cut off (Millipore, Billerica, MA). Filtered samples were analyzed by HPLC (Model 5600A CoulArray multi-electrode detector), concentrations were determined from a standard curve, and were normalized to the 100nM DHBA internal standard.

### Plasma corticosterone analysis

In order to determine whether physiological stress responses differed after contextual re-exposure in males exposed to either direct social defeat (intruders) or witness stress, plasma corticosterone was measured using a Corticosterone ELISA (Enzo Life Sciences, Farmingdale, NY). Plasma was diluted 1:40 and run according to manufacturer protocol. Plates were read using a Synergy 2 Multi-Mode plate reader (Bio Tek, Winooski, VT) with Gen5 software (Bio Tek, Winooski, VT).

### TIMP-1 analysis in plasma

As an index of cardiac remodeling, a MilliPlex Rat CVD Panel 1 kit (EMD Millipore, Billerica, MA) was run to quantify tissue inhibitor of matrix metalloproteinases-1 (TIMP-1). Plasma was diluted 1:2 in assay buffer and run in duplicate according to manufacturer protocol. The multiplex was read and analyzed using a Bio-Rad Bio-Plex Luminex SD system (Hercules, CA).

### Cytokine quantification in plasma and brain tissue

A 10-Plex Bio-Plex Assay (Bio-Rad Laboratories, Hercules CA) was used according to manufacturer protocol and our previous report [12] to determine the effect of intruder vs. witness stress on peripheral inflammatory status. Data were normalized to control (% control) in order to account for large interkit variability. Cytokine levels in LC homogenates were determined using a Quantikine Rat IL-1β/IL-1F2 ELISA (R&D Systems, Minneapolis, MN) per the manufacturer protocol. The plate was read using a Synergy 2 Multi-Mode plate reader (Bio Tek, Winooski, VT) with Gen5 software (Bio Tek, Winooski, VT) and expression was normalized to total protein concentration obtained with a Pierce BCA Protein Assay (Thermo Scientific, Rockford, IL).

### High Mobility Group Box-1 (HMGB1) western blot analysis

HMGB1 protein levels in LC brain homogenates were determined by western blot analysis, and normalized to a GAPDH loading control. 20μg of each homogenate was separated by SDS-PAGE, transferred to a PVDF membrane, and blocked using Blok fluorescent blocker (Millipore, Billerica, MA). The PVDF membranes were incubated with rabbit anti-HMGB1 (1:5000) and mouse anti-GAPDH (1:1000; Abcam, Cambridge, MA) overnight at 4°C. The secondary antibodies goat anti-rabbit Dylight 680 and goat anti-mouse Dylight 800 (Thermo Fisher, Waltham, MA) were diluted 1:5000. HMGB1 and GAPDH bands were quantitated using LI-COR Odyssey Image Studio software (LI-COR, Lincoln, NE).

### Statistical analysis

Statistically significant outliers were identified and removed using the Modified Thompson Tau outlier test. All cardiovascular data were subjected to a standard Two-Way ANOVA (α = 0.05) to determine effects of stress and time. Separate Two-Way ANOVAs were conducted to identify significant differences between groups during the stress/control exposure and that of the home cage recovery on days 1, 5 and 11. All other data sets were analyzed using a standard One-Way ANOVA to determine effects of stress on each endpoint. Tukey’s post-hoc analysis was used for both One- and Two-Way ANOVAs using GraphPad Prism 6 (La Jolla, CA) and SAS JMP 10 (Cary NC) software.

## Results

### Cardiovascular response to social defeat and witness stress

#### Blood pressure and heart rate during stress/control exposure

**Fig 2:**
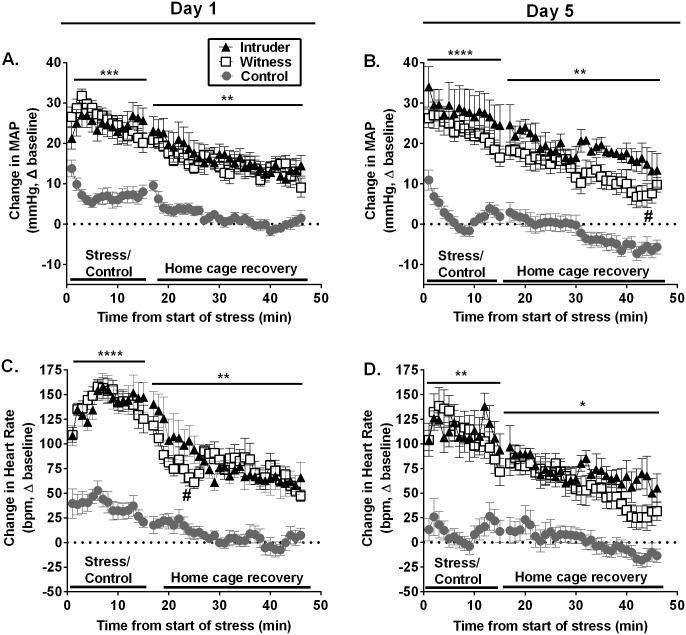
Hemodynamic and tachycardic response to social defeat and witness stress on the first and fifth exposure to stress. Physical interaction with the resident in the form of social defeat (intruder) and witnessing such an interaction produced nearly identical increases in mean arterial pressure (MAP) on days 1 (A) and 5 (B) of stress exposure above that of controls. Similarly, the heart rate (HR) response was also equally elevated in intruder and witness groups on days 1 (C) and 5 (D) of stress, significantly above that of controls. Upon being returned to their home cage, MAP and HR exhibited a time dependent decreases during the 30-minute home cage recovery period. *p<0.05, **p<0.01, ***p<0.001, ****p<0.0001 intruders/witnesses vs. controls; There were two instances during the home cage recovery where the cardiovascular response in witnesses recovered more quickly than intruders (^#^p<0.05 witnesses vs. intruders).

In order to determine the cardiovascular effects of intruder (physical social defeat) or witness (psychological) stress, continuous cardiovascular recordings were obtained during the first and fifth stress exposure. Surprisingly these data indicated that both direct physical interaction with the resident (intruders) and witnessing this social defeat exposure produced nearly identical increases in MAP during the 15-minute stress exposure on days 1 (effect of stress: F_(2, 330)_ = 380.2, p<0.0001; Fig 2A) and 5 (effect of stress: F_(2, 300)_ = 458.4, p<0.0001; effect of time: F_(29, 567)_ = 2.908, p<0.0001; Fig 2B). During the 30-minute home cage recovery period, both intruder and witness groups demonstrated a moderate reduction in MAP over time upon being returned to the home cage (effect of time day 1: F_(29, 602)_ = 5.452, p<0.0001; effect of time day 5: F_(14, 300)_ = 2.383, p<0.01), yet MAP for both intruder and witness groups remained significantly elevated above controls throughout the recovery period (effect of stress day 1: F_(2, 602)_ = 661.3, p<0.0001; effect of stress day 5: F_(2, 567)_ = 514.3, p<0.0001, Fig 2A and 2B). Analogous to the findings in MAP, both witness and intruder groups exhibited nearly identical stress-induced tachycardic responses during stress exposure on both day 1 (effect of stress: F_(2, 315)_ = 552.6, p<0.0001; Fig 2C) and day 5 (effect of stress: F_(2, 300)_ = 279.4, p<0.0001, Fig 2D). Furthermore, stress-induced increases in HR of both witness and intruder groups declined across the 30-minute home cage recovery period on both day 1 (interaction: F_(58, 583)_ = 1.436, p<0.05, Fig 2C) and day 5 (effect of time: F_(29, 597)_ = 3.252, p<0.0001), but remained significantly elevated above controls (day 5 effect of stress: F_(2, 597)_ = 333.3, p<0.0001; Fig 2D). Importantly, neither stress group exhibited habituation to the sympathomimetic response of social stress over the five-day repeated exposure period. Specifically, witness stressed rats pressor and tachycardic responses were comparable between days 1 and 5 of stress exposure (effect of day MAP: F_(1, 70)_ = 3.246, p = 0.076; effect of day HR: F_(1, 65)_ = 0.4036, p = 0.527) and intruder rats exhibited an overall increased MAP response from day 1 to day 5 (effect of day: F_(1, 35)_ = 4.863, p<0.05). In contrast, analysis of control animals did suggest cardiovascular habituation to handling occurred from days 1 to 5 (effect of day MAP: F_(1, 100)_ = 6.976, p<0.01; effect of day HR: F_(1, 100)_ = 12.30, p<0.001). Together these data indicate that both direct physical contact with the resident and witnessing the social defeat produces nearly identical increases in MAP and HR, that do not habituate across the 5 day stress period.

#### Stress-induced cardiac arrhythmias

**Fig 3:**
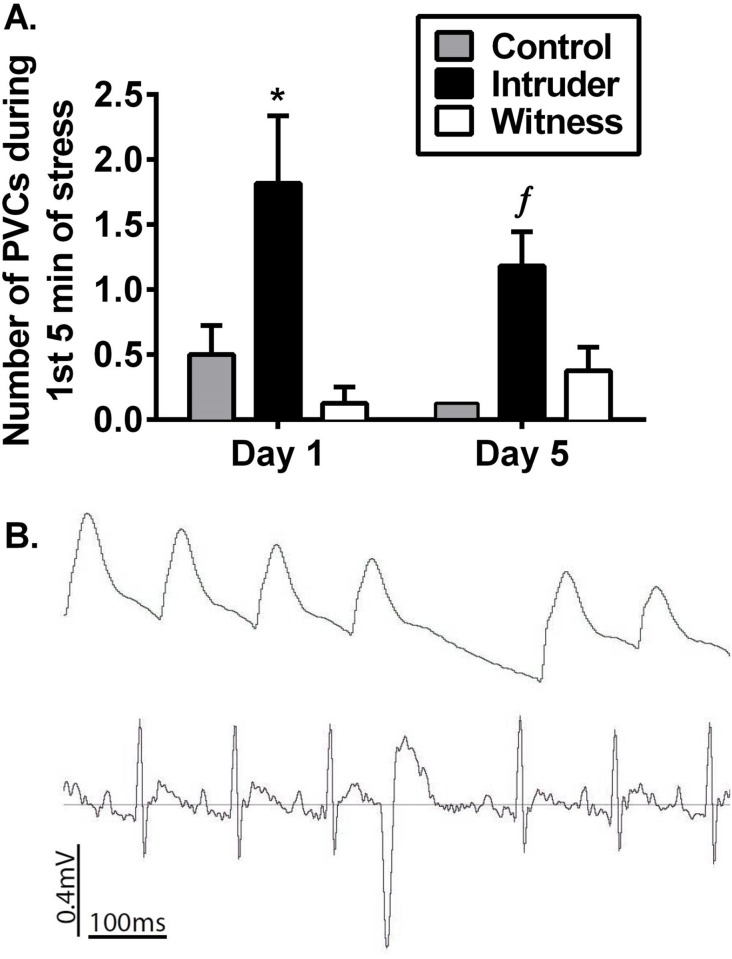
The effect of social defeat and witness stress on Premature Ventricular Contractions (PVCs). Social defeat stress exposure on days 1 and 5 produced an increased incidence of PVCs in intruder rats compared with both control and witness exposure (A). PVCs were determined by analyzing electrical activity within the ECG trace and the corresponding blood pressure traces (B). *p<0.05 vs. control+witness rats; ^ƒ^p = 0.053 vs. control.

Stress-induced increases in BP and HR have been associated with an increased incidence of arrhythmias [17–19]; therefore, in the present study the number of PVCs that occurred on days 1 and 5 of stress or control exposure was assessed. Analysis of PVCs detected from ECG traces during the first 5 minutes of stress exposure indicated a main effect of stress (F_(2, 44)_ = 9.566, p<0.001; Fig 3A), such that intruders exhibited a greater number of PVCs compared to witness and control rats. These data were surprising considering that intruders and witness stressed rats displayed comparable hemodynamic and tachycardic responses during the first 5 minutes of witness exposure. This difference is likely due to the nature of the stressor encountered since the number of PVCs during the baseline (resting) period did not differ between the two stress groups on either day 1 or day 5 (effect of stress: F_(2, 23)_ = 1.137, p = 0.34).

#### Cardiovascular sensitivity to stress context re-exposure

**Fig 4:**
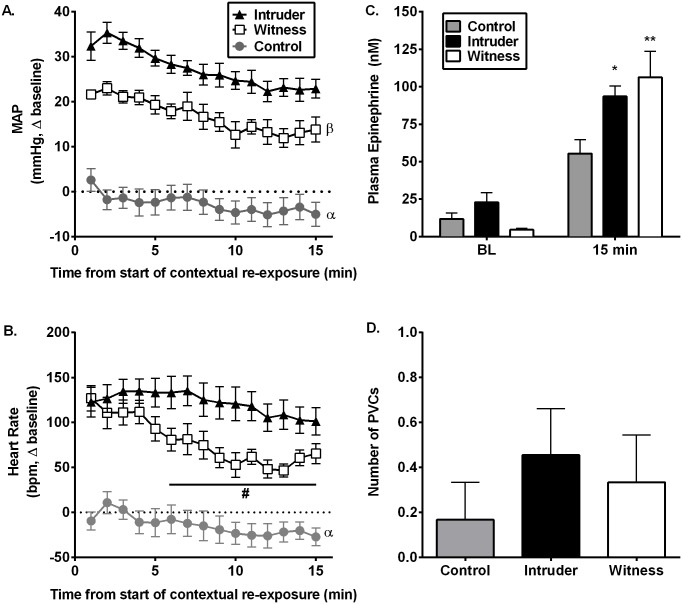
Cardiovascular and epinephrine responses to contextual re-exposure. Contextual re-exposure 6 days after the fifth and final social stress or control exposure produces robust increases in mean arterial pressure (MAP; A) and heart rate (HR; B) in both intruder and witness rats (^α^p<0.0001 control vs. intruders and witnesses across exposure). Interestingly, intruder rats had a more robust hemodynamic response to being placed in a resident’s empty cage compared with witnesses (^β^p<0.0001 witnesses vs. intruders across exposure) and the tachycardic response was more prolonged than in witness stressed rats (^#^p<0.05 witness vs. intruders). However, HPLC analysis of plasma indicates that both intruders and witnesses exhibit enhanced circulating epinephrine during this re-exposure (C). Surprisingly, this robust increase in MAP and HR was not associated with an increase in the number of premature ventricular contractions (D) that occurred during the first 5 minutes. *p<0.05, **p<0.01 vs. controls.

In order to determine whether a history of exposure to social defeat or witness stress alters the subsequent cardiovascular response to re-exposure to the social stress environment (an empty cage), we subjected all rats to a contextual re-exposure 6 days after the final witness/intruder/control manipulation. This 15-minute contextual re-exposure produced robust increases in MAP (effect of stress: F_(2, 315)_ = 496.1; p<0.0001; Fig 4A) and HR (effect of stress: F_(2, 299)_ = 369.9; p<0.0001; Fig 4B) in both the intruder and witness groups as compared with controls. Strikingly, while both intruders and witnesses showed nearly identical increases in MAP and HR during days 1 and 5 of social stress (Fig 2), during context re-exposure, intruders exhibited greater increases in MAP compared to both witness and control rats (Fig 4A). Alternatively, the tachycardic response to the stress context re-exposure was comparable between intruder and witness rats during the first 5 minutes; however, witnesses exhibited a faster HR recovery (Fig 4B). Furthermore, in all rats the increased MAP and HR during the re-exposure period were also associated with enhanced plasma epinephrine levels (interaction: F_(2, 25)_ = 4.468, p<0.05, Fig 4C). Importantly, this effect was more robust in rats with a history of social defeat or witness stress exposure.

To identify whether cardiovascular adaptation or sensitization occurred throughout the repeated exposures to control, intruder, or witness stress, the cardiovascular response within a stress group was compared during stress between days 1, and 5 (Fig 2) and day 11 (Fig 4). Control and witness rats demonstrated blunted MAP and HR response to context re-exposure compared to their cardiovascular response on days 1 and 5 during control or witness stress, respectively (Control effect of day MAP: F_(2, 100)_ = 38.16, p<0.0001; control effect of day HR: F_(2, 145)_ = 17.07, p<0.0001; witness effect of day MAP: F_(2, 70)_ = 5.72, p<0.0001; witness effect of day HR: F_(2, 99)_ = 3.706, p<0.05). Alternatively, intruders demonstrated similar pressor responses during context re-exposure in the absence of the resident compared with day 5 of social defeat, and an even greater increase in MAP compared to that of the first day of social defeat (effect of day: F_(2, 55)_ = 8.123, p<0.001). Furthermore, the HR response during re-exposure in intruder rats remained comparable to the tachycardic effect during social defeat on either days 1 or 5 (effect of day: F_(2, 60)_ = 2.046, p = 0.138). However, unlike social stress-induced increases in PVCs that occurred in intruders during social defeat, context re-exposure did not produce PVCs in any stress group (F_(2, 20)_ = 0.4692, p = 0.6; Fig 4D), further indicating the importance of the resident’s presence in the cage for the induction of arrhythmias in intruder rats.

#### Chronic changes in resting cardiovascular measures

**Fig 5:**
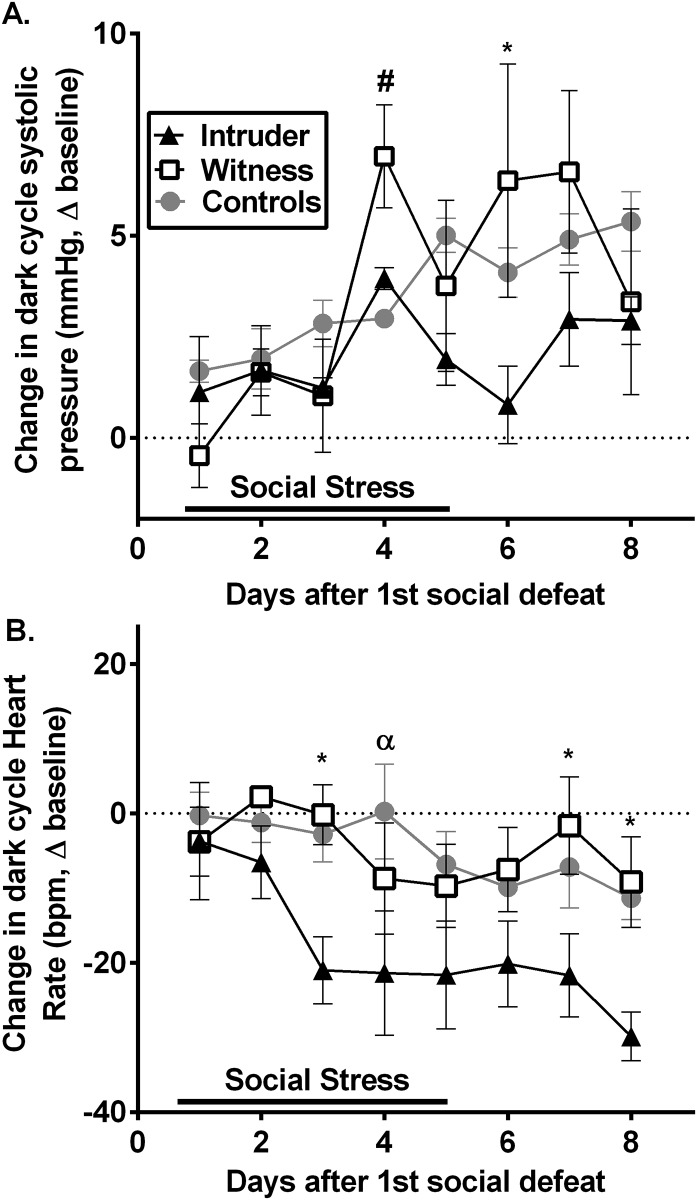
The long-term changes in resting blood pressure and heart rate induced by social stress exposure. Dark cycle systolic pressure increased across the duration of the study (A). This effect was moderately enhanced in witness rats on days 4 and 6 compared to control and intruder rats, respectively. Conversely, heart rate (HR) measured during the dark cycle was significantly reduced in intruder rats across the duration of the study, compared to both witness and control animals (B). *p<0.05 witness vs. intruder; ^#^p<0.05 witness vs. control; ^α^p<0.05 control vs. intruder.

Analysis of persistent changes in resting dark cycle/active systolic BP and HR indicated that systolic BP, in general, increased across the duration of the study (effect of time: F_(7, 115)_ = 2.557, p<0.05; Fig 5A). Witness stressed rats exhibited greater increases in systolic BP on days 4 (F_(2,13)_ = 4.9, p<0.05) and 6 compared to control and intruder rats, respectively. Conversely, intruders demonstrated greater reductions in HR compared to both witness and control animals (effect of stress: F_(2, 119)_ = 14.62, p<0.0001; Fig 5B), which persisted for the duration of the study (effect of time: F_(7, 119)_ = 2.337, p<0.05). These data suggest that intruder and witness stress produce differential long-term cardiovascular consequences. Importantly, stress did not result in an increase in locomotor activity during the dark cycle (F_(2, 137)_ = 1.393, p<0.252; see S4 Fig). Therefore, it is unlikely that altered activity levels were driving these observed effects.

The transient increase in BP that was observed in witness rats was not associated with long-term cardiac remodeling as analysis of ventricular weights was not significantly different between stress groups (F_(2, 34)_ = 0.8863, p = 0.422; Average ventricular weight (g)/100 g Body weight ± SEM (n): control: 0.2895 ± 0.001 (11); intruder: 0.2926 ± 0.001 (18); witness: 0.3069 ± 0.011 (8)). However, immediately following the 15-minute context re-exposure, rats previously exposed to witness stress exhibited significant increases in TIMP-1 levels, an early biomarker of increased cardiovascular disease risk [43–47], as compared with both control and intruder rats (effect of stress: F_(2, 23)_ = 7.7, p<0.01; Average pg/mL ± SEM (n)): control: 12,272 ± 1419 (11); intruder: 10157 ± 1758 (8); witness: 23,317 ± 4140 (7)).

### Social defeat and witness stress produce differential effects on inflammation

We and others have previously indicated that social defeat stress produces alterations in the immune system resulting in enhanced central [12, 13, 48] and peripheral inflammation [12, 13, 32, 49]. Importantly, neuroinflammation has been shown to mediate neuronal activity [50, 51] and in the context of the LC, the major source of norepinephrine to the brain, has been shown to mediate depressive-like behaviors [12, 13] and cardiovascular control [52–58]. Surprisingly, analysis of IL-1β in the LC indicated that neither intruder nor witness stressed rats exhibited enhanced neuroinflammation compared to controls 6 days after the final social stress exposure (effect of stress: F_(2,11)_ = 0.57, p = 0.58; Table 1). However, HMGB1, a marker of neuroimmune priming [59], demonstrated an increase in the LC of both intruders and witnesses compared to controls. Due to exaggerated variability in the stress groups however, this effect did not reach significance (F_(2, 19)_ = 3.300, p = 0.059; Table 1). This suggests that although there were no differences in resting LC IL-1β, intruder and witness stress could have produced enduring changes in the inflammatory system within the LC through the upregulation of HMGB1. A more extensive evaluation of the neuroinflammation at additional stress-related time points and brain regions is warranted.

**Table 1:** Inflammatory-related protein concentrations in the LC and plasma in response to contextual re-exposure.

Analyte	Control	Intruder	Witness		Tukey’s post-hoc
**Locus coeruleus (LC)**					
**IL-1β (pg/mL)**	**213.6 ± 22.7(4)**	**192.7 ± 14.8(5)**	**186.7 ± 17.6(5)**		**N.S.**
**HMGB1/ GAPDH protein**	**134.8 ± 36.8(8)**	**248.3 ± 62.7(8)**	**380.4 ± 100.1(6)**		**N.S.**
**Plasma**					
**IL-2 (%Control)**	**100.0 ± 13.4(6)**	133.1 ± 10.45(8)*		**88.7 ± 6.1(6)**	* p<0.05 vs control/witness
**IL-4 (%Control)**	**100.0 ± 16.3(6)**	127.1 ± 10.8(8)*		**79.8 ± 8.0(6)**	* p<0.05 vs. witness
**MCP-1 (%Control)**	**100.0 ± 6.0(6)**	131.7 ± 8.9(8)*		**124.2 ± 10.5(5)**	* p<0.05 vs. control
**IL-6 (%Control)**	**100.0 ± 10.8(6)**	**112.7 ± 11.45(8)**		**99.5 ± 15.4(6)**	**N.S.**
**INF-γ (%Control)**	**100.0 ± 13.2(6)**	**124.2 ± 14.2(8)**		**96.9 ± 15.2(6)**	**N.S.**

All data are represented as the mean ± SEM with sample size (n). N.S. = nonsignificant p>0.05.

**Fig 6:**
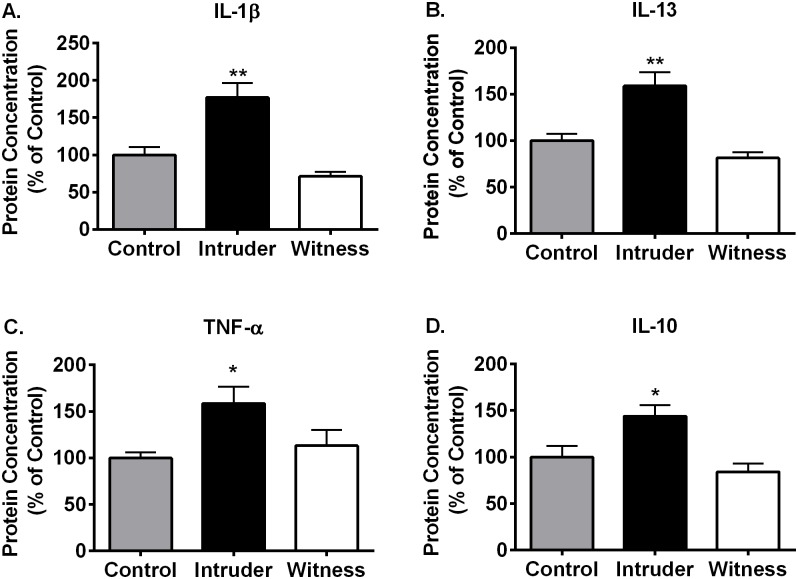
The effect of contextual re-exposure on plasma cytokine levels of control, intruder, and witness stressed rats. Intruder rats exhibit greater proinflammatory IL-1β (A), IL-13 (B), TNF-α (C), and anti-inflammatory IL-10 (D) in response to contextual re-exposure 6 days after the final stress exposure compared to both witnesses and control rats. *p<0.05, **p<0.01, ***p<0.001 intruders vs. controls and witnesses.

The peripheral inflammatory system, on the other hand, indicated robust effects of stress for several pro- and anti-inflammatory cytokines following the 15-minute contextual re-exposure (Fig 6, Table 1). Specifically, plasma from intruder rats exhibited significantly elevated IL-1β (effect of stress: F_(2, 17)_ = 14.09, p<0.001, p<0.05; Fig 6A), IL-13 (effect of stress:F_(2, 17)_ = 13.13, p<0.001; Fig 6B), tumor necrosis factor alpha (TNF-α; effect of stress: F_(2, 17)_ = 4.077, p<0.05; Fig 6C), and IL-10 (effect of stress: F_(2, 17)_ = 8.013, p<0.01; Fig 6D) compared with both witness and control rats. This same trend was also evident for the proinflammatory cytokine IL-2 (effect of stress: F_(2, 17)_ = 5.134, p<0.05) and anti-inflammatory cytokine IL-4 (effect of stress: F_(2, 17)_ = 4.064, p<0.05), while IL-6 (effect of stress: F_(2, 17)_ = 0.3802, p = 0.69) and interferon gamma (INF-γ; effect of stress: F_(2, 17)_ = 1.156, p = 0.34) remained largely unchanged (see Table 1). Interestingly, monocyte chemoattractant protein (MCP)-1 was elevated in both intruder and witness groups (effect of stress: F_(2, 16)_ = 3.696, p<0.05); however, this effect only reached statistical significance in the intruder group (Table 1). Importantly, these effects were not due to physical injury in the intruders. Throughout the duration of the study only two intruders sustained minor lacerations, each was treated with antibiotic ointment, and the peripheral cytokine response for each of these animals fell within the normal range of the rest of the intruder group.

### Social defeat and witness stress produce differential effects on behavior but not stress-related physiological endpoints

In order to determine if social defeat or witness stress produce differential effects on motivational behavior, rats were subjected to the sucrose preference test. There were no pre-existing differences in sucrose preference (F_(2, 18)_ = 2.51, p = 0.11), yet surprisingly, social stress only produced mild reductions in sucrose preference in witness stressed rats (effect of stress: F_(2, 17)_ = 4.96, p<0.05; Table 2), indicating that under these conditions witness stress, but not 15 minutes of social defeat paired with a witness, may produce significant anhedonic-like behavior. Importantly, these effects were evident 1 hour into the sucrose preference test, but were no longer significant after 3 hours of sucrose exposure (F_(2, 25)_ = 1.442, p = 0.26), suggesting these behavioral effects were modest and transient. Furthermore, the lack of anhedonic-like behavior in the intruder group was not due to ineffective defeats. Intruders were consistently attacked (average number of attacks ± SEM: 4.85 ± 0.71, n = 8) and displayed supine submissive postures (average latency to submit (sec) ±SEM: 421.58 ± 40.57, n = 8) in response to daily defeats by a novel resident each day. In addition, both intruders and witnesses exhibited significant adrenal hypertrophy compared with controls (effect of stress: F_(2, 32)_ = 14.13, p<0.0001) and plasma corticosterone levels during contextual re-exposure (effect of stress: F_(2, 15)_ = 16.08, p<0.001; Table 2). While absolute body weight, body weight gain, and splenic weight remained unaffected (Table 2), the adrenal and corticosterone data suggest that witnesses and intruders, regardless of the emergence of behavioral disturbances, are likely demonstrating a hyperactivity of the hypothalamic-pituitary-adrenal (HPA) axis.

**Table 2:** Stress-related behavioral and physiological effects of control, witness, and social defeat exposure.

	Control	Intruder	Witness	Tukey’s post-hoc
**1 hour Sucrose Preference (%)**	**87.00 ± 2.6(9)**	**81.48 ± 2.6(6)**	71.4 ± 4.7(6)*	* p<0.05 vs. control
**3 hour Sucrose Preference (%)**	**85.30 ± 3.18(10)**	**83.20 ± 4.73(6)**	**71.67 ± 6.51(6)**	**N.S.**
**Adrenal Weight/ 1000 g Body Weight**	**0.06 ± 0.01(12)**	0.11 ± 0.01(8)***	0.10 ± 0.01(8)**	**p<0.01, *** p<0.001 vs. control
**Plasma Corticosterone (ng/mL)**	**36.55 ± 2.13(6)**	169.10 ± 20.64(6)***	202.30 ± 31.68(6)****	***p<0.001, ****p<0.0001 vs. control
**Absolute Body Weight**	**424.40 ± 8.5(12)**	**421.00 ± 10.56(8)**	**422.80 ± 14.5(8)**	**N.S.**
**Body Weight Gain**	**132.80 ± 19.5(11)**	**100.00 ± 10.99(8)**	**90.7 ± 10.8(6)**	**N.S.**
**Splenic Weight/ 1000 g Body Weight**	**2.05 ± 0.1(12)**	**2.11 ± 0.14(8)**	**2.20 ± 0.1(8)**	**N.S.**

All data are represented as the mean ± SEM with sample size (n). N.S. = nonsignificant p>0.05.

## Discussion

While it has long been established that the physical altercation of social defeat produces robust sympathomimetic effects in intruder rats [17–19, 60], this is the first report where, within the same cage, the cardiovascular effects of witnessing a social defeat are directly compared to the physical exposure of social defeat. Interestingly, the current study shows that while both intruders and witnesses exhibited nearly identical stress-induced increases in MAP and HR, physical (social defeat) versus psychological (witness) social stress exposure produced distinctly different effects. For example, daily exposure to witnessing social stress produced transient increases in resting systolic BP and modest depressive-like anhedonia, while intruders displayed only reductions in resting HR. Alternatively, what was seemingly a sub-threshold duration of stress in intruders, in fact rendered intruders more sensitive to a context re-exposure stress as they exhibited a more robust sympathomimetic and immune response when compared with witnesses and controls. Together, these findings indicate that physical versus psychological exposure to social defeat stress produces distinct long-term cardiovascular, behavioral, and immune consequences.

Since the emergence of the resident-intruder paradigm, the behavioral and physiological ramifications of social defeat exposure in the intruder have been well characterized. Consistent with previous findings [17–19, 60], the present study determined that direct contact with the resident during social defeat exposure was sufficient to induce acute, stress-induced elevations in BP and HR, that persisted throughout the duration of the defeat and recovery periods. Yet this study builds on these findings to reveal that direct contact with the resident is not required to produce the significant sympathomimetic effects responsible for the robust pressor and tachycardic responses to social defeat. Based on the physical nature and relative stress intensity of social defeat, we anticipated that direct physical exposure to social defeat would elicit a greater sympathetic activation compared to the psychological nature of witness stress. Therefore, one of the most striking findings of this study is that witnesses exhibit nearly identical stress-induced pressor and tachycardic responses to that of intruders. Interestingly, neither witnesses nor intruders exhibited habituation to their respective social stress conditions, as both groups mounted similar or enhanced sympathomimetic and tachycardic responses to stress upon repeated exposures. This lack of habituation may be central to social stress as other predictable homotypic stressors, such as repeated restraint, have been shown to result in physiological habituation of not only HPA activity but also cardiovascular responses including HR and BP [61]. In contrast to the strikingly similar social stress-induced tachycardic and pressor responses exhibited between witness and intruder rats, PVCs occurred selectively in the intruder group, suggesting that it is the combined interaction with the resident rather than absolute increases in HR and/or MAP that is responsible for inducing the emergence of PVCs. This is further supported by the findings that the intruders no longer displayed PVCs during context re-exposure despite exhibiting a comparable magnitude of tachycardia and blood pressure, further highlighting the necessity of the physical interaction between the resident and intruder for generating arrhythmias.

Social stress exposure is well recognized to increase the incidence of cardiovascular disease [10, 11] and therefore, this study also evaluated the long-term changes in resting cardiovascular parameters. Interestingly, enduring changes in the cardiovascular system were distinctly different between witness stressed rats and socially defeated intruders. In the intruder group, there were persistent decreases in resting HR while systolic BP remained largely unchanged. This chronic reduction in HR may likely represent an adaptive compensatory mechanism induced by the baroreceptor reflex, shifting sympathetic and parasympathetic activity in order to maintain BP. Unlike intruders, witnesses exhibited significant elevations in resting systolic BP while HR remained stable across the duration of the study. These findings are consistent with our previous work and others revealing that social stress produces reductions in heart rate variability [18, 62], supporting a shift towards greater sympathetic control. However, increased pressure without a compensatory reduction in HR, as seen in witness stressed rats, may indicate impaired baroreflex sensitivity, specifically the threshold at which an increase in BP produces a compensatory reduction in HR. In fact, social defeat stress in rodents has been reported to impair baroreflex sensitivity, such that larger increases in BP are required to initiate this reflex [63]. Interestingly, this reduction in baroreflex sensitivity has also been shown to occur in humans in response to stress exposure with a more pronounced reduction in individuals undergoing psychological stress compared to those experiencing physical stress [64]. Therefore, quantification of both baroreflex sensitivity and parasympathetic-sympathetic balance in witness and intruder rats will be an important next step and have the potential to reveal stress specific mechanisms of chronic cardiovascular regulation.

In humans, increased levels of Timp-1 have been shown to be associated with indices of cardiovascular remodeling and major cardiovascular risk factors [44, 65, 66]. In fact, it has been suggested that Timp-1 may be an early marker of increased incidence of major adverse cardiac events [43, 44]. Importantly, although socially stressed rats did not demonstrate ventricular hypertrophy, witness stressed rats exhibited robust increases in Timp-1 over that of controls and intruder rats. Together these data suggest that although large scale cardiovascular remodeling did not occur 6 days after the final stress/control exposure, witness stressed rats may have an increased risk for developing subsequent cardiovascular disease including atherosclerosis, cardiomyopathy, congestive heart failure, myocardial infarction, and aortic aneurysm [45–47, 67–69].

While the long-term cardiovascular consequences of stress exposure differed based on the specific stress manipulation, both intruders and witnesses exhibited significant adrenal hypertrophy, and enhanced circulating plasma corticosterone levels. Interestingly, despite eliciting a physiological stress response regardless of physical contact with the resident, 15 minutes of exposure to the resident in the presence of a witness was not sufficient to induce decreases in motivational behavior in the intruder as we have previously identified to occur following a 30-minute social defeat exposure [12–14, 70]. Similar paradigms to the one used in this study that indicate a significant effect of direct social defeat stress, included a segment of time behind the partition in the resident’s cage [29, 30, 33], an aspect not included in the present study. It is therefore possible that the emergence of depressive-like behavioral responses in intruders results from the combination of the physical (social defeat) and psychological (behind partition time) aspects of this social defeat model. Alternatively, it is possible that the presence of a witness could buffer the psychological impact of social defeat; a similar phenomenon has been noted in nociceptive and stress studies conducted in both humans and rodents [71–73]. Yet, what is a sub-threshold social defeat in an intruder rat that is physically interacting with the resident is sufficient to induce an anhedonic-like behavior in rats that bear witness to this stress. Additional studies to further characterize intruder behavior in the presence and absence of a witness could provide valuable insight into the conflicting reports of intruder behavior. Taken together, the current study suggests that it may be the psychological rather than the physical aspect of this stress that plays a greater role in the development of stress-induced behavioral/motivational alterations.

The LC-noradrenergic system is a major node of integration between neural control of behavioral and cardiovascular function as it is known to exert control over the cardiovascular system [52–58] and has long been implicated in the emergence of depression [74–76]. We have previously shown that exposure to social defeat can produce long lasting increases in resting inflammation within the LC [12, 13], which may function to increase neuronal activation [77], suggesting that LC neuroinflammation could drive the negative behavioral and cardiovascular repercussions associated with social stress. We went on to demonstrate that neuroinflammation was a critical determinant of social stress-induced anhedonia [12, 13]. Thus, in the present study we hypothesized that LC neuroinflammation may be elevated in witness stressed rats. It is important to note that brain tissue used to measure cytokine levels in this study was collected immediately following the 15-minute resident cage context re-exposure. Therefore at this time point, these levels are reflective of the resting state because IL-β in the brain peaks between 3 and 24 hours after stress or immune challenge [78–81]. Resting levels of neuroinflammation within the LC of witness or intruder stressed rats did not differ significantly from that of controls. These data again support the notion that this 15-minute exposure to social defeat in intruder rats was in some ways a sub-threshold exposure, since 30 minutes of social defeat stress in intruders is sufficient to induce increases in resting neuroinflammation [12, 13].

HMGB1 is a protein released from neurons, microglia, and astrocytes [59, 82, 83] in response to a variety of stressors [59, 84] and is thought to contribute to neuroinflammatory priming and perpetuation of proinflammatory release through several mechanisms including signaling through Toll-like receptors [59, 85, 86]. Our data indicate that although resting levels of cytokines in the LC are not increased following context re-exposure, both social defeat (physical) and witness (psychological) stressed animals had increased HMGB-1 expression in the LC compared to controls. While HMGB1 secretion occurs readily after stress exposure, it requires at least 1 hour to translocate into the cytoplasm of neurons to begin the proinflammatory response [84]. Therefore, it is likely that both intruders and witnesses would exhibit enhanced proinflammatory cytokine release in the LC at a later time point.

Inflammatory priming is not a unique phenomenon of the brain, but is also well recognized to occur in the periphery. Prior history with stress or lipopolysaccharide primes the inflammatory response such that future challenges result in an enhanced and more rapid stress-induced cytokine release from macrophages or splenic cells [12, 87–89]. Therefore, if there is a history of stress exposure, plasma cytokine release rises significantly within 15 minutes and peaks by one hour signifying the onset of stress-induced responses [40, 90, 91]. We have previously shown that rats undergoing 5 daily exposures to 30 minutes of social defeat do not exhibit changes in resting circulating cytokines 5 days after stress, but they do exhibit enhanced splenic cytokine release upon stimulation with concanavalin A, suggesting inflammatory priming occurred in CD4+ spleen cells selectively within a susceptible subset of socially defeated rats [12]. Therefore, in the present study, we hypothesized that rats with a history of social defeat (intruders) would likely exhibit enhanced cytokine release in response to a subsequent stress exposure. In support of this hypothesis, plasma cytokines were significantly elevated only in intruder rats in response to the contextual re-exposure. However, without cytokine measurements at multiple time points we cannot rule out the possibility that a slightly delayed cytokine response was also evident in witness-exposed rats. It is important to note that this phenomenon was not due to injuries incurred by the intruders during social defeat exposure, but rather suggests distinct differences in the immune consequences between physical versus psychological social stress.

### Limitations and future directions

This study provides valuable insight into the cardiovascular, behavioral, and inflammatory consequences of direct social defeat versus witness stress. However, there are some limitations that have been considered. It has been demonstrated that exposure to an unknown environment or unknown conspecific are inherently stressful [92]. In the present study, the effects of exposure to the novel environment of the resident’s cage or a male conspecific have not been independently evaluated and therefore it cannot be ruled out that these factors, in part, contribute to the repercussions of witness stress. However, it should be noted that a similar model conducted in mice determined that 10 days of sensory exposure to an aggressive resident in the absence of social defeat had no effect on social interaction [31]. Furthermore, witnessing social defeat stress also had no effect if the partition between the witness and the social defeat episode was opaque. Taken together, these data suggest that it is the exposure to the social defeat episode, rather than novelty, that drives the behavioral alterations in response to bearing witness to a social stress episode. Furthermore, the current study represents an initial characterization of the cardiovascular and neuroinflammatory consequences of direct social defeat versus witness stress. As such, it would be interesting to determine the longevity of the exhibited cardiovascular effects. Similarly, while the LC is well suited to study stress-induced behavioral and cardiovascular deficits, and has been the focus of neuroinflammatory changes following social defeat [12, 13], analysis of other relevant stress sensitive brain regions, such as the prefrontal cortex, hippocampus, amygdala, and dorsal raphe, would provide greater insight into potential differential neuroinflammatory effects of these stressors.

## Conclusion

Based on the findings of this study, both social defeat (physical) and witness (psychological) stressors are capable of eliciting robust cardiovascular responses acutely, which can result in long term cardiovascular dysregulation if adaptive processes do not engage this system to return to rest. Interestingly, although social stress-induced cardiovascular responses of both intruders and witnesses are similar, social defeat and witness stress produced opposing behavioral and inflammatory consequences. Therefore, this ethologically relevant model provides valuable insight into the respective contributions of both the physical and psychological aspects of social stress in the development of depression and associated cardiovascular and inflammatory diseases. Based on the experimental design, this paradigm could be used to study witness stress in females. Currently few animal models of female social stress exist and in many cases do not allow for the direct comparison between males and females. Development of such a model is especially important as females are more sensitive to stress [93, 94] and are more likely to develop depression [95–97] and stress-induced cardiovascular disturbances [98, 99]. Therefore, continuing to validate this model in females will be an important advance in understanding the mechanisms involved in enhanced stress sensitivity in females.

## Supporting information

S1 Fig Schematic of social defeat/witness stress with resident cage dimensions. The resident cage where social defeat/witness stress occurred measures 8.5”x8.5”x16”. During stress exposure the Plexiglas partition was placed into the resident cage creating an 8.5”x8.5”x4” compartment for the witness (W) and an 8.5”x8.5”x12” compartment for the resident and intruder (R+I).
(TIF)

S2 Fig Schematic of CV telemetry set up. (A) Receivers were placed behind witness (W) and intruder (I) cages during chronic 24-hour/day and pre-defeat baseline cardiovascular measurements to simultaneously collect cardiovascular data from each cage. (B) During defeat, receivers were placed under the resident (R) cage to measure stress-induced heart rate and blood pressure simultaneously in paired witnesses and intruders.
(TIF)

S3 Fig Histological verification of LC punch placement. Since the LC is a small brain region, histological verification of punch placement was performed to ensure accurate placement of each LC sample obtained. 30μm pre-punch (A) and post-punch (B) slices were obtained, dehydrated, and stained with neutral red. Stained slides were then viewed under a microscope to ensure proper punch placement and depth. Punches were compared to the Paxinos and Watson Brain Atlas at Bregma -10.20 mm (C).
(TIF)

S4 Fig Relative activity during the dark cycle. In order to ensure that transient increases in dark cycle systolic blood pressure and reductions in dark cycle heart rate were not due to changes in witness and intruder activity, respectively, relative dark cycle activity was calculated as a change from each rat’s pre-stress baseline relative level of activity. Baseline consisted of dark cycle averages obtained 2 days prior to the start of stress/control. Exposure to either direct social defeat (intruders) or witness stress did not result in a shift in dark cycle activity (F_(2, 137)_ = 1.393, p<0.252) compared to controls. Therefore, it is unlikely that altered dark cycle activity was driving the transient increases in dark cycle systolic blood pressure and reductions in heart rate exhibited by witnesses and intruders, respectively. Note, a relative value of 1 denotes no change from baseline activity.
(TIF)

S1 File Additional methods are provided for social defeat/witness stress and in vivo cardiovascular telemetry. (PDF)
