# Supplementary figures and images for: Physical versus psychological social stress in male rats reveals distinct cardiovascular, inflammatory and behavioral consequences
Source: PLoS One. 2017 Feb 27;12(2):e0172868. doi: 10.1371/journal.pone.0172868 (PMC5328366; doi:10.1371/journal.pone.0172868)

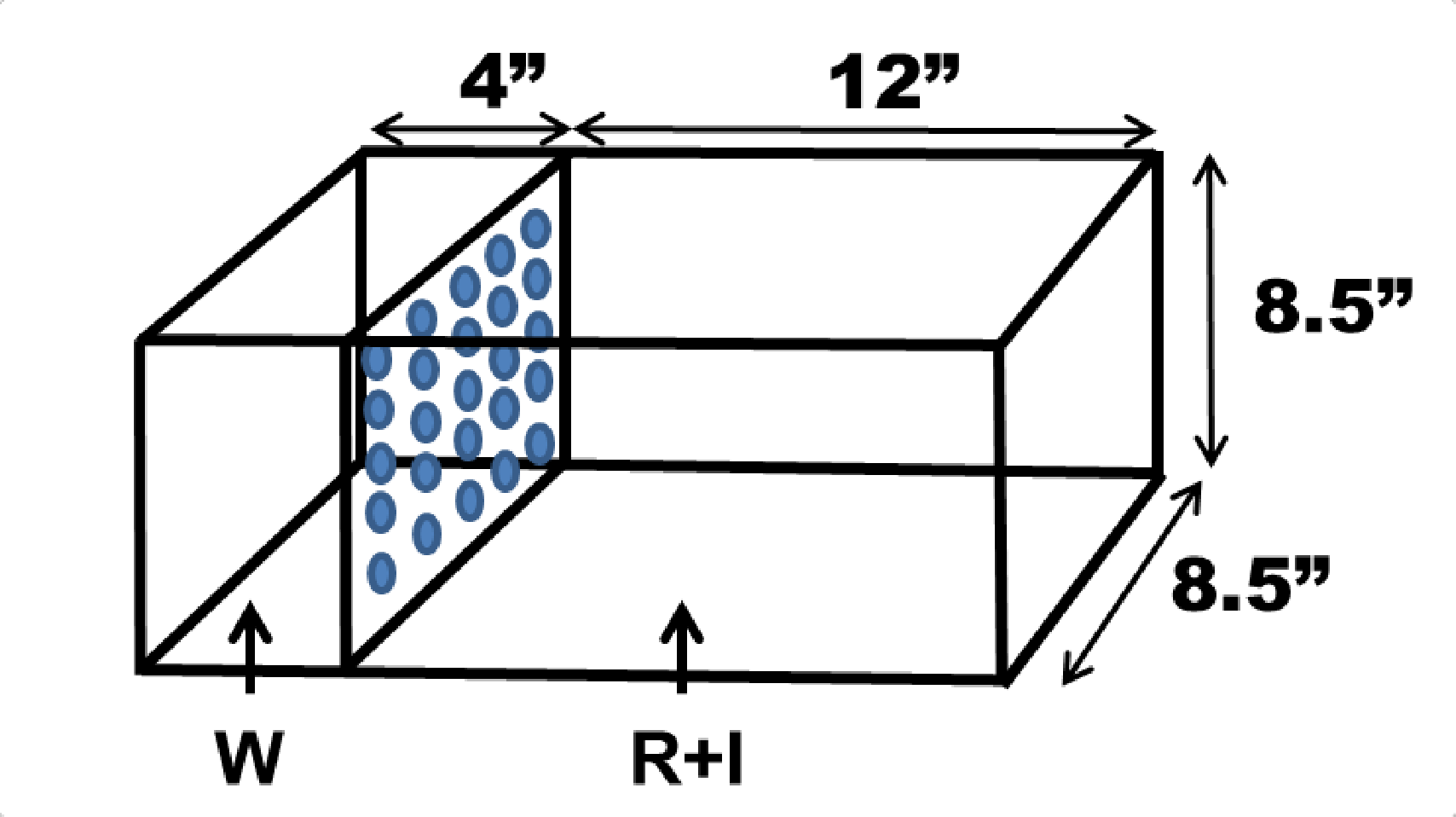

Supplement: S1 Fig — The resident cage where social defeat/witness stress occurred measures 8.5”x8.5”x16”. During stress exposure the Plexiglas partition was placed into the resident cage creating an 8.5”x8.5”x4” compartment for the witness (W) and an 8.5”x8.5”x12” compartment for the resident and intruder (R+I). (TIF) [file pone.0172868.s001.tif]

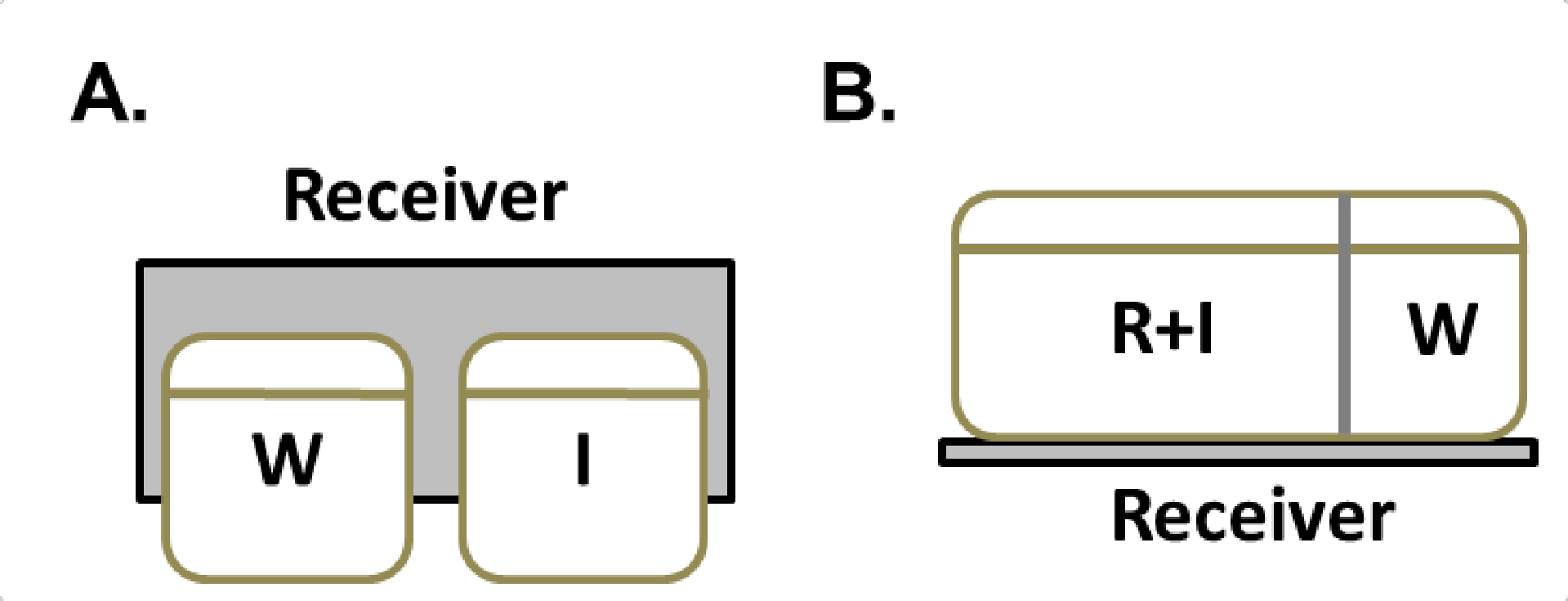

Supplement: S2 Fig — (A) Receivers were placed behind witness (W) and intruder (I) cages during chronic 24-hour/day and pre-defeat baseline cardiovascular measurements to simultaneously collect cardiovascular data from each cage. (B) During defeat, receivers were placed under the resident (R) cage to measure stress-induced heart rate and blood pressure simultaneously in paired witnesses and intruders. (TIF) [file pone.0172868.s002.tif]

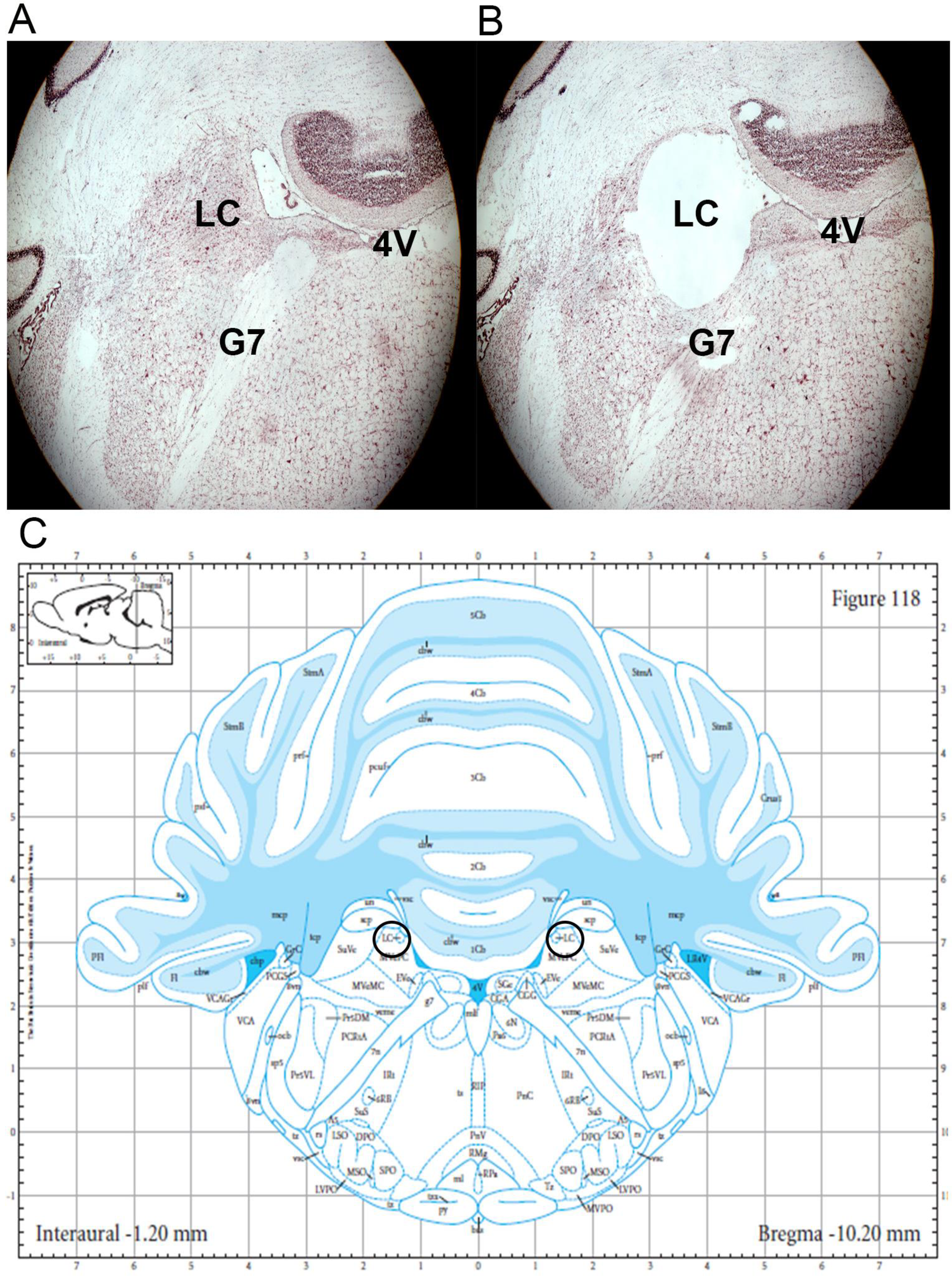

Supplement: S3 Fig — Since the LC is a small brain region, histological verification of punch placement was performed to ensure accurate placement of each LC sample obtained. 30μm pre-punch (A) and post-punch (B) slices were obtained, dehydrated, and stained with neutral red. Stained slides were then viewed under a microscope to ensure proper punch placement and depth. Punches were compared to the Paxinos and Watson Brain Atlas at Bregma -10.20 mm (C). (TIF) [file pone.0172868.s003.tif]

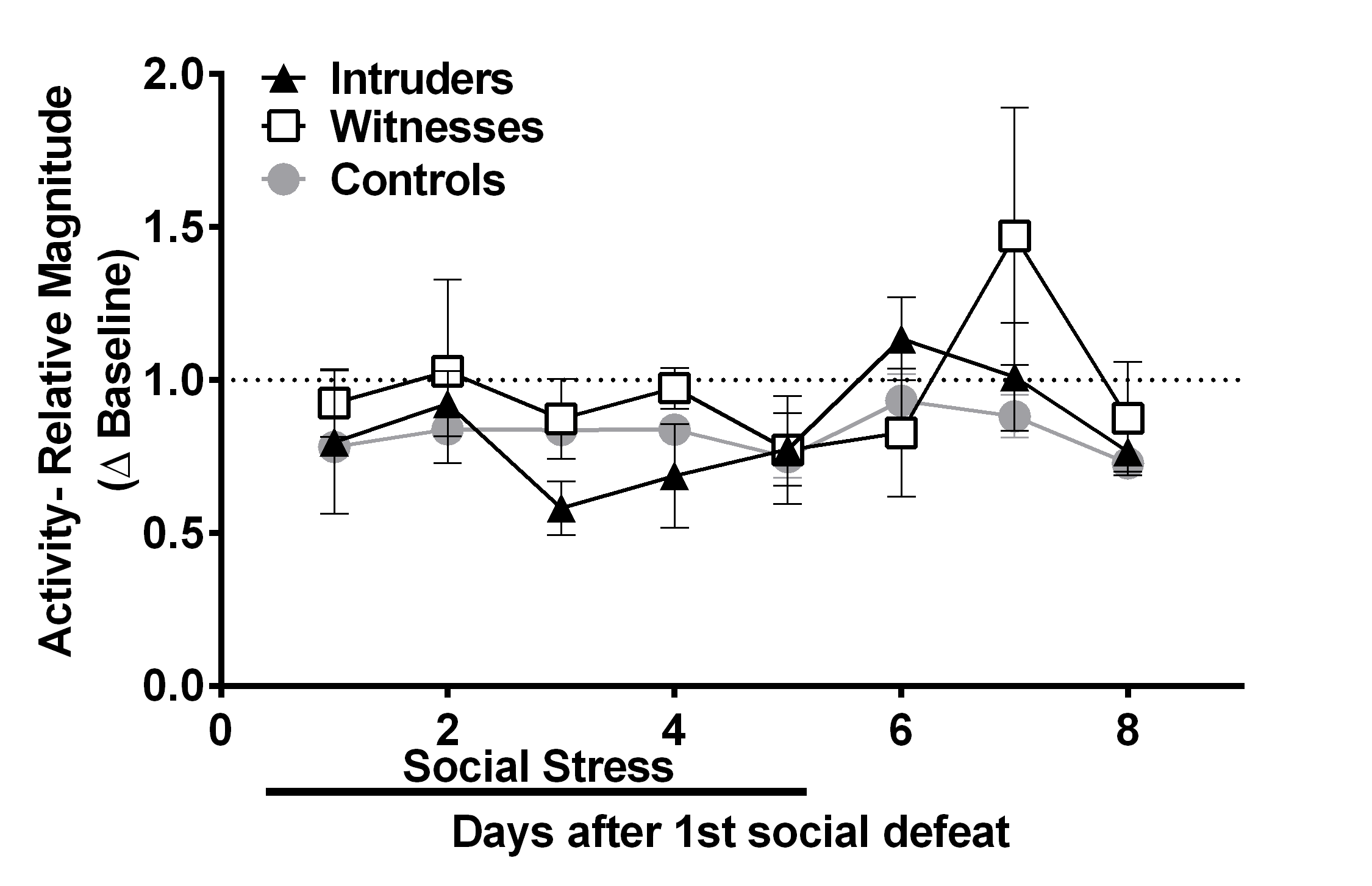

Supplement: S4 Fig — In order to ensure that transient increases in dark cycle systolic blood pressure and reductions in dark cycle heart rate were not due to changes in witness and intruder activity, respectively, relative dark cycle activity was calculated as a change from each rat’s pre-stress baseline relative level of activity. Baseline consisted of dark cycle averages obtained 2 days prior to the start of stress/control. Exposure to either direct social defeat (intruders) or witness stress did not result in a shift in dark cycle activity (F(2, 137) = 1.393, p<0.252) compared to controls. Therefore, it is unlikely that altered dark cycle activity was driving the transient increases in dark cycle systolic blood pressure and reductions in heart rate exhibited by witnesses and intruders, respectively. Note, a relative value of 1 denotes no change from baseline activity. (TIF) [file pone.0172868.s004.tif]
