# Supplementary material for: Physical versus psychological social stress in male rats reveals distinct cardiovascular, inflammatory and behavioral consequences
Source: PLoS One. 2017 Feb 27;12(2):e0172868. doi: 10.1371/journal.pone.0172868 (PMC5328366; doi:10.1371/journal.pone.0172868)
Supplement: S1 File — (PDF) [file pone.0172868.s005.pdf]

Physical versus psychological social stress in male rats reveals distinct cardiovascular, inflammatory and behavioral consequences

Finnell JE, Lombard CM, Padi AR, Moffitt CM, Wilson LB, Wood CS, Wood SK.

Supporting Information:

Methods

Section A. Social Defeat/Witness Stress

This animal model was modified from the version developed by Sial et al. 2016 [1] and Warren et al. 2013 [2] using the rat model of social defeat developed Miczek [3]. Long-Evans retired breeders were screened for their level of aggression prior to being included in the study. Inclusion criteria consisted of 1) exhibiting an attack latency of less than 60 seconds, 2) total number of attacks ≥ 4 within the first 5 minutes of day 1, and 3) effective attacks that did not result in injury to the intruder. Sprague-Dawley rats were randomly assigned into "intruder", "witness", or "control" groups and singly housed for the duration of the study. During stress, witnesses were paired with the same intruder for each of the 5 stress exposures and intruder/witness pairs were exposed to a different Long-Evans retired breeder for 15 minutes during each of the 5 consecutive days. This truncated social defeat paradigm was used in order to protect intruders from incurring injuries during defeat as this could confound our subsequent inflammation data. Witnesses were placed in the resident cage for the duration of the 15 minute stress period behind a Plexiglas partition containing 70 mm diameter holes to allow for olfactory, visual, and auditory cues. Intruders spent the entire 15-minute stress period directly interacting with the resident. Resident cage dimensions and schematic of social defeat/witness stress are shown in S1 Fig. Behavioral responses of the resident, intruder, and witnesses were recorded each day of the social defeat period. Intruders and witnesses were returned to their respective home cages daily following social stress. Control animals were not present in the room during social defeat exposures and

control manipulation consisted of brief handling for no more than 15 seconds/day, to mimic the duration of time each witness and intruder was handled by the experimenter.

Section B*. In vivo* cardiovascular telemetry

The current study utilized a Ponemah telemetry system (version 6.30) in conjunction with HD-S11 F2 and F0 transmitters to simultaneously record real time ECG and blood pressure of paired intruder and witness stressed rats. A schematic of the layout of the telemetry system during social defeat/witness stress and at rest is depicted in S2 Fig. At rest, dual transmitter DSI receivers were placed behind the appropriate witness and intruder home cages as shown in S2 Fig. A. This set up was used to collect the chronic 24 hour/day and pre-defeat baselines, collecting from two separate cages at a time. During defeat/witness stress, receivers were placed underneath the resident cage, as shown in S2 Fig. B, for continuous during defeat/witness stress cardiovascular recordings. Based on this set up, a maximum of 12 animals could be run at a time (n=6 witnesses paired with n=6 intruders). Control animals were run in a separate cohort. Cardiovascular transmitter receivers were placed below the cage of each control, similarly to what is depicted in S2 Fig. B, for all cardiovascular measurements as dual recordings were not required.

Literature Cited

1. Sial OK, Warren BL, Alcantara LF, Parise EM, Bolanos-Guzman CA. Vicarious social defeat stress: Bridging the gap between physical and emotional stress. Journal of neuroscience methods. 2016;258:94-103.
2. Warren BL, Vialou VF, Iniguez SD, Alcantara LF, Wright KN, Feng J, et al. Neurobiological sequelae of witnessing stressful events in adult mice. Biol Psychiatry. 2013;73(1):7-14.
3. Miczek KA. A new test for aggression in rats without aversive stimulation: differential effects of d-amphetamine and cocaine. Psychopharmacology Berlin. 1979;60:253-9.

4. Paxinos G, Watson C. The Rat Brain in Stereotaxic Coordinates. 6 ed. London: Academic Press; 2007.
